# Puberty stages of primary school students

**DOI:** 10.1186/1687-9856-2013-S1-P82

**Published:** 2013-10-03

**Authors:** Aditya Suryansyah

**Affiliations:** 1 Endocrinology Unit, Harapan Kita Women and Children Hospital, Jakarta, Indonesia

**Keywords:** Puberty, primary school

## Background

Puberty is the transition from child to adult. Signs of puberty begin 8-13 years of age in girls and 9-14 years in boys. Puberty begins with breast budding in girls and enlargement of the testes in boys.

## Purpose

Knowing the early signs of Puberty in primary school students in South Tangerang, Indonesia.

## Method

This is a descriptive cross sectional study of 4th-6th grade to students in two primary schools in South Jakarta, in January 2011.

## Results

There are 471 samples met the inclusion criteria, 215 boys and 256 girls. In girls, signs of puberty at age of 9 - <10 years are 48.2%, while in age 12 - <13 all had puberty. In boys, signs of puberty at age of 9 - <10 years are 1.7%, while in age 12 - <13 years are 66.7%. Pubic hair sign in girls at the age of 9 - <10 years (4.4%), in boys aged 11 - <12 years (29%). Occurred menarche at age 10 - <11 years (5.7%).

## Conclusion

Signs of puberty in two primary school students in South Tangerang has occurred in the age of 9 - <10 years, both women and men. At the age of 12 - <13 entered puberty had all girls, while boys are not in all (66.7%). In women, the pubic hair appear earlier than men. Menarche occurred from children aged 10 - <11 years.

